# Sociodemographic disparities in the establishment of health records among 0.5 million migrants from 2014 to 2017 in China: a nationwide cross-sectional study

**DOI:** 10.1186/s12939-021-01584-2

**Published:** 2021-12-02

**Authors:** Jun Wang, Jingmin Zhu, Xueyao Wang, Yue Che, Yang Bai, Jue Liu

**Affiliations:** 1 Center for Health Policy Research and Evaluation, Renmin University of China, Beijing, 100872 China; 2 Department of Economics, University of Birmingham, B15 2TT, Birmingham, UK; 3 Department of Epidemiology and Biostatistics, School of Public Health, Peking University Health Science Centre, Beijing, 100080 China; 4 Institute for Global Health and Development, Peking University, Beijing, 100871 China; 5 Key Laboratory of Reproductive Health, National Health Commission of the People’s Republic of China, Beijing, 100083 China; 6 School of Public Health, Peking University; Institute for Global Health and Development, Peking University, Beijing, 100191 China

**Keywords:** Migrants, China, Sociodemographic disparities, Health record, Health services utilization

## Abstract

**Background:**

Migrants account for a large part of China’s population. Many policies and inventions have been taken to improve access to public health services and the health of migrants. China’s Basic Public Health Services(BPHS) are a series of public health services in this policy domain, which aims at promoting the access of public health sevices and improve health equity of residents. The establishment of health records is the fundamental service of BPHS. However, there is little known about the establishment of health records among migrants in China, which hinders the more efficient provision of health services for migrants, and health equity is difficult to achieve. Based on the research gap, this study aims at showing the sociodemographic disparities in the establishment rate of health records, and identifying priorities and recommendations for promoting health equity of migrants in China.

**Methods:**

This study used national data from China Migrants Dynamic Survey (CMDS) from 2014 to 2017 to evaluate the sociodemographic disparities in the establishment rate of health records and utilization of relevant public health services. The study included 539,926 respondents. Following the descriptive statistics of migrants, we showed the establishment rate of health records by sociodemographic characteristics and migrating related characteristics. Multivariate analysis was conducted to explore the associations between sociodemographic charicteristics, migrating related charicteristics and the establishment of health records.

**Results:**

The establishment rate of health records among migrants in the sampled years were 22.99, 38.44, 27.29% respectively, and 29.18% in general, and there existed heterogeneity in the establishment rate of health records by sociodemographic charicteristics and migrating related charicteristics. Female migrants who were older, from middle age, married or living with partner, with higher educational attainment, with urban household registration, migrated for longer time, migrated for the reason of studying or family issues, migrated in province were more likely to establish health records.

**Conclusion:**

There existed sociodemographic disparities in the establishment rate of health records and inequalities in the utilization of health records services among migrants in China. Migrating related characteristics also had impact on the establishment status. Policies should take both supply side and demand side of health services to improve the health equity of migrants, which means that relative departments should continue to invest in primary healthcare centers to improve their ability to provide services as well as migrants’ health literacy.

**Supplementary Information:**

The online version contains supplementary material available at 10.1186/s12939-021-01584-2.

## Introduction

Migrants are a large population in China, and the equity in the utilization of health services of migrants is an important policy issue as well as academic issue. According to the Communiqué of the Seventh National Census of China, as of November 1, 2020, the number of migrants in China reached 375.8 million, accounting for 26.03% of the total population. Compared with the data of the sixth national census in 2010, the number of migrants increased by 154.4 million, an increase of 69.73%. The migrants have made a huge contribution to China’s economic development and economic growth. Especially since the Reform and Opening-up in 1978, a large number of rural residents have entered the cities, which has promoted China’s urbanization and industrial upgrading, and has improved their ability to support families on a microscopic level and promotes the accumulation of social wealth on a macro level.

The Chinese government has always attached great importance to the health of the migrants. Beginning in 2009, the Chinese government began to provide all the Chinese people with free basic public health services to promote the equity of health services and further improve the health of residents, which is called Basic Public Health Services (BPHS) [1, 2]. This project requires all primary healthcare centers to provide basic public health services for all residents in the jurisdiction, including registered population and migrants [3]. Since 2009, the items of basic public health services have continuously increased, and the compensation for each person served by primary healthcare centers has also increased. Supplemental Table 1 shows the relevant information about China’s BPHS items and funding [4].

Many scholars have conducted researches on BPHS provided for the migrant population in China. Since the development of the east, middle and west regions are thought to be different, the differences of BPHS’s utilization of migrants in different regions in China has been studied. For example, Zhang et al.(2017) showed that internal migrants in more developed eastern regions used less public health services [5]. Zhu et al.(2019) focused on health education, one of the items of BPHS, and their study showed that as far as HIV education, there existed significant regional disparities among migrants in China [6]. On a broader scale, the utilization of health services of migrants in different regions in China were also significantly different, which may hinders the improvement of accessibility of health services [7–10].

Many studies focus on the fact that many residents migrated from rural areas to urban areas, which may have an impact on their utilization of health services, as there are more adequate health resources in urban areas [11, 12]. The utilization of BPHS in rural areas are relatively low [13]. After moving to urban areas, the health literacy of migrants from rural areas has been improved and this promoted their participation in health education activities [14, 15]. The impact of the special household registration system on the utilization of BPHS in China has also been researched. Compared with migrants with rural household registration, migrants with urban household registration had a higher likelihood to utilize BPHS [5, 7, 16, 17]. Other factors were also explored in the studies of this research domain, such as social integration, age, ethnicity, employment status, current health situation, medical insurance coverage, rage of migrating, family income were also associated with the utilization of BPHS [7, 13, 16, 18–21].

Although residents don’t need to pay for any services, and the government has launched various policies to improve the accessibility of BPHS, the utilization of the services is still insufficient. In the study of Guo et al.(2019), only 33.9% of the participants received chronic disease education [22]. Tang et al. (2021) found only 36.2% of migrant older adults receiving free physical examinations, which is an important item of BPHS [23]. The improvement of utilization of BPHS has become an important policy issue.

Of all the items of BPHS, establishing health records is an important item of China’s BPHS. It is essential for understanding the health status of the migrants and better providing health services to residents. It is also the basis for providing other BPHS for the migrants. Some researchers has investigated associated factors of establishing health records, and older female migrants with homeownership, long-term settlement intention, medical insurance, having more than three local friends, were employed were more likely to establish health records [5, 18–20, 24, 25]. In terms of the effect of employment status and household income, there existed heterogeneity in current research [13, 21, 24]. However, we found that there is still a lack of researches on sociodemographic disparities in the establishment rate of health records of migrants in China. To address this knowledge gap, we used national data from China Migrants Dynamic Survey (CMDS) to evaluate sociodemographic disparities in the establishment of health records among 0.5 million migrants from 2014 to 2017 in China.

## Methods

### Study design and data source

This is a nationwide cross-sectional study in China. Data was collected by 2014, 2016, 2017 waves of CMDS in China. The three waves of CMDS were conducted annually by China’s Health Commission, China population and development research center, Chinese center for disease control and prevention, Health Commission of 31 provinces and Xinjiang Production and Construction Corps in mainland China. The national survey aimed at understanding the living condition and public services utilization of migrants, enhancing the efficiency of related policies. CMDS used a probability proportional to size (PPS) sampling method which is a stratified, multi-stage and proportional scale sampling. The survey covered 436 cities and counties in mainland China. The participants of the survey were migrants aged above 15 years whose household registration is not at the current residence, and had resided in the place for more than a month for working or living. All the participants received an informed consent.

### Data collection

The questionnaire of the survey is designed uniformly, including sociodemographic information, occupation, willingness to migrate or resident and public services utilization. All provinces carried out face-to-face survey with smart phones or pads installed with a specially developed interview system. All the participants are directly interviewed by investigators with unified training. The sample contained 539,926 participants.

### Establishment rate of health records

The primary outcome in the present study is the establishment rate of health records. The migrants were asked the question: Have you established the health record at the current community? (yes, no, or not sure). The establishment rate of health records is calculated by dividing the number of participants who have established health records (answering yes) by the total number of participants.

### Associated factors

#### Sociodemographic characteristics

We included six sociodemographic characteristics: sex (female or male), age (65 years and above, 55–64 years, 45–54 years, 35–44 years, 25–34 years, 25–34 years, or 15–24 years), regions (east, middle, or west), marital status (married/living with partner or never married/ divorced/widowed), educational attainment (above college degree, college, high school and equivalent or middle school and below), and household registration status (urban or rural).

#### Migrating related characteristics

We included three migrating related characteristics: length of migration (11 years and above, 6–10 years or 0–5 years), reasons of migration (working/business or studying/family issues), and rage of migration (out of province or in province).

### Statistical analysis

Statistical analyses were carried out by using Stata version 16.0 (StataCorp LLC. Texas, USA). Sociodemographic information and the establishment rate of health records were showed using descriptive statistical analysis. The associated factors of the establishment status of health records were investigated by performing binary logistic regression and using the odd ratio (OR) and 95% confidence intervals (CIs). A two-side *p* value less than 0.05 was considered as statistically significant.

## Results

### Basic characteristics of migrants

A total of 539,926 migrants were included in this study, 200,937 in 2014, 16,900 in 2016, and 169,989 in 2017, respectively. Table 1 shows the characteristics of the sampled population. The percentage of male participates were higher than female of for the sampled years, and 54.38% were male of all the migrants. The age range of the most participates were from 15 to 44 of the three years. The east region had the most participates for three years which takes account of more than half of the sampled population, while the middle region had the least participates. 79.55% of the migrants were married or living whit partner. More than 60% migrants obtained an education of middle school or less. More than 80% of the migrants’ household registration status was agricultural, with a percentage of 83.72% in general. 69.46% of the migrants had stayed at the destination place for 5 or less years. As the reasons of migration, 85.29% of the migrants came to the destination to work or do businesses.

**Table 1:** Sociodemographic characteristics and migrating related characteristics among migrants in China, Migrants Population Dynamic Monitoring Survey 2014, 2016 and 2017 (*N* = 539,926)

	2014		2016		2017		Overall	
	N	%	N	%	N	%	N	%
**Sociodemographic characteristics**								
**Sex**								
male	117,647	58.55	88,088	52.12	87,871	56.69	293,606	54.38
female	83,290	41.45	80,912	47.88	82,118	48.31	246,320	45.62
**Age**								
15–24	40,062	19.94	26,648	15.77	23,906	14.06	90,616	16.78
25–34	77,057	38.35	65,709	38.88	65,473	38.52	208,239	38.57
35–44	56,747	28.24	45,193	26.74	44,741	26.32	146,681	27.17
45–54	24,036	11.96	23,566	13.94	26,651	15.68	74,253	13.75
55–64	3035	1.51	5659	3.35	6499	3.82	15,193	2.81
65-			2225	1.32	2213	1.30	4438	0.82
**Region**								
East	101,984	50.75	82,000	48.52	86,995	51.18	270,979	50.19
Middle	33,986	16.91	29,000	17.16	28,999	17.06	91,985	17.04
West	64,967	32.33	58,000	34.32	53,995	31.76	176,962	32.78
**Marital status**								
Never married/ Divorced/widowed	48,006	23.89	31,929	18.89	30,472	17.93	110,407	20.45
Married/living with partner	152,931	76.12	137,071	81.11	139,517	82.07	429,519	79.55
**Educational attainment**								
<=Middle school	133,812	66.59	104,296	61.71	103,186	60.70	341,294	63.21
High school or equivalent	41,289	20.55	37,682	22.30	37,224	21.90	116,195	21.52
College	25,183	12.53	26,214	15.51	28,687	16.88	80,084	14.83
>College	653	0.32	808	0.48	892	0.52	2353	0.44
**Household registration status**								
Agricultural	170,904	85.05	140,441	83.10	140,687	82.76	452,032	83.72
Nonagricultural	30,033	14.95	28,559	16.90	29,302	17.24	87,894	16.28
**Migrating related characteristics**								
**Length of migration**								
0–5	153,208	76.25	115,601	68.40	106,203	62.48	375,012	69.46
5–10	28,177	14.02	30,435	18.01	34,681	20.40	93,293	17.28
> = 11	19,552	9.73	22,964	13.59	29,105	17.12	71,621	13.26
**Reasons of migration**								
other	23,855	11.87	27,711	16.40	27,872	16.40	79,438	14.71
Working or business	177,082	88.13	141,289	83.60	142,117	83.60	460,488	85.29
**Rage of migration**								
In province	98,534	49.04	85,977	50.87	86,199	50.71	270,710	50.14
Out of province	102,403	50.96	82,860	49.03	83,790	49.29	269,053	49.83

Notes: missing data, age, 506 (0.09%); rage of migration,163 (0.03%)

### Sociodemographic disparities in the establishment of health records among migrants

In full sample, the establishment rate of health records among migrants were 22.99,38.44,27.29% respectively, and 29.18% in general. Of all the groups, there existed a sharp increase of the rate followed by a decline in 2017. Table 2 shows the establishment rate of health records among migrants by sociodemographic characteristics and migrating related characteristics. The establishment rate of health records among female migrants is higher than that of male migrants. The gap between the two groups was the largest in 2016.The participants aged 65 and above reported highest establishment rate of health records, 45.71% of 2016,35.96% of 2017 and 40.43% in general. The participants aged 15 to 24 had the lowest portion of establishing health record(19.82% of 2014, 34.33% of 2016,27.70% of 2017 and 26.09% in general). Among the three regions, the migrants of west region showed the highest rates of establishing health record (28.33%), followed by the middled region (28.03%) and the east region (17.91%) in 2014. Then in 2016 and 2017, the middle region showed the highest rates (53.53,43.23% respectively), and the east region had the lowest portion (30.57,25.52% respectively). Migrants who were married or living with partner had the higher rate of establishing health records than those who were never married, divorced, or widowed. Migrants who had college degree had the highest rates in 2014 and 2017 and migrants who had education above college had the highest rate in 2016. In terms of household registration status, the finding shows that migrants with urban household had higher establishment rate of health records.

**Table 2:** Establishment rate of health records among migrants in China of 2014, 2016 and 2017 by sociodemographic and migrating related characteristics

	Establishment rate of health records			
	2014 N (%)	2016 N (%)	2017 N (%)	Overall N (%)
**Total**	46,186(22.99)	64,957(38.44)	46,389(27.29)	157,532(29.18)
**Sociodemographic characteristics**				
**Sex**				
Male	26,117(22.20)	32,228(36.59)	23,010(28.92)	81,355(28.52)
Female	20,069(24.10)	32,729(40.45)	23,379(31.17)	76,177(31.85)
**Age**				
15–24	7940(19.82)	9149(34.33)	5359(27.70)	22,448(26.09)
25–34	18,064(23.45)	26,097(39.72)	18,570(31.04)	62,731(30.96)
35–44	13,724(24.19)	17,573(38.88)	12,653(30.17)	43,950(30.55)
45–54	5696(23.70)	8818(37.42)	7064(28.58)	21,578(29.84)
55–64	762(25.11)	2303(40.70)	1799(29.42)	4864(32.85)
> = 65		1017(45.71)	944(35.96)	1961(40.43)
**Region**				
East	18,403(17.91)	24,366(30.57)	14,789(25.52)	57,558(24.14)
Middle	9525(28.03)	15,525(53.53)	11,518(43.23)	36,568(40.80)
West	18,258(28.33)	25,066(42.01)	20,082(30.03)	63,406(33.42)
**Marital status**				
Never married/ divorced/widowed	9270(19.31)	10,461(32.76)	6663(26.21)	26,394(25.05)
Married or living with partner	36,916(24.14)	54,496(39.76)	39,726(30.76)	131,138(31.29)
**Educational attainment**				
<=Middle school	30,271(22.62)	38,595(37.01)	26,967(28.83)	95,833(28.90)
High school or equivalent	9630(23.33)	15,134(40.16)	10,586(31.33)	35,350(31.35)
College	6130(24.35)	10,884(41.52)	8617(32.62)	25,631(32.94)
>College	155(23.74)	344(42.57)	219(25.86)	718(31.11)
**Household registration status**				
Rural	38,444(22.50)	53,291(37.95)	37,368(29.39)	129,103(29.44)
Urban	7742(25.78)	11,666(40.85)	9021(32.88)	28,429(33.05)
**Migrating related characteristics**				
**Length of migration**				
0–5	33,497(21.87)	44,228(38.26)	27,442(30.22)	105,167(29.25)
6–10	7386(26.21)	11,947(39.25)	10,633(30.66)	29,966(32.12)
> = 11	5303(27.13)	8782(38.24)	8314(28.57)	22,399(31.28)
**Reasons of migration**				
Other	6700(28.09)	12,043(43.46)	8933(34.22)	27,676(35.63)
Working or business	39,486(22.30)	52,914(37.45)	37,456(29.15)	129,856(29.06)
**Rage of migration**				
In province	26,674(27.07)	39,075(45.45)	27,424(34.40)	93,173(35.26)
Out of province	19,512(19.06)	25,818(31.16)	18,965(25.33)	64,295(24.72)

Notes: missing data, rage of migration,64(0.01%)

### Establishment rate of health records among migrants by migrating related characteristics

As far as the groups by migration characteristics, in 2014, there was a tendency that migrants who migrating for longer time has higher rate of establishing health record. Then in 2016 and 2017,migrants who had migrated for 6 to 10 years had highest rate of establishing health records (39.25,30.66% respectively). Compared with migrants who had migrated for the reason of working or business, migrants who migrated for other reasons had higher portion of establishing health record. Migrants who had migrated in province had higher rate than those who had migrated out of province.

### Factors related to health record enrollment status for migrants

In the multivariable model 1, sex, age, region, marital status, educational attainment, household registration status, length of migration, reasons of migration, rage of migration were associated with the establishment rate of health records, after adjusting for potential factors (Table 3). Compared with migrants who age 15 to 24, migrants aged 25–34 years (aOR = 1.10; 95% CI,1.07–1.12), migrants aged 35–44 years (aOR = 1.08; 95% CI,1.06–1.11), migrants aged 45–54 years (aOR = 1.05; 95% CI,1.02–1.08), migrants aged 55–64 years (aOR = 1.14; 95% CI,1.10–1.19), and migrants aged 65 and above (aOR = 1.48; 95% CI,1.39–1.57) were more likely to establish health record. Compared with migrants whose destination place is the west region, migrants headed to the middle region (aOR = 1.28; 95% CI,1.26–1.30) were more likely to establish health record. Compared with migrants who were never married, divorced, or widowed, those who were married or living with partner (aOR = 1.36; 95% CI,1.34–1.39) were more likely to establish health record. Compared with migrants who received middle school education and below, migrants whose education level is high school or equivalent (aOR = 1.13; 95% CI,1.11–1.15), College (aOR = 1.24; 95% CI,1.21–1.26), above college (aOR = 1.25; 95% CI,1.14–1.37) had the high possibility to establish health records. Compared with migrants with rural household registration, migrants with urban household (aOR = 1.09; 95% CI,1.07–1.11) registration had a higher likelihood to establish health records.

**Table 3:** Factors related to health record enrollment status for migrants

	Univariable analysis		Multivariable analysis			
			Model 1		Model 2	
	cOR(95% CI)	*P*	aOR(95% CI)	*P*	aOR(95% CI)	*P*
**Sex**						
male	1(Ref)		1(Ref)		1(Ref)	
female	1.17(1.16–1.19)	< 0.001	1.14(1.12–1.15)	< 0.001	1.21(1.19–1.24)	< 0.001
**Age**						
15–24	1(Ref)		1(Ref)		1(Ref)	
25–34	1.27(1.25–1.30)	< 0.001	1.10(1.07–1.12)	< 0.001	1.12(1.09–1.14)	< 0.001
35–44	1.25(1.22–1.27)	< 0.001	1.08(1.06–1.11)	< 0.001	1.12(1.09–1.15)	< 0.001
45–54	1.21(1.18–1.23)	< 0.001	1.05(1.02–1.08)	< 0.001	1.10(1.07–1.14)	< 0.001
55–64	1.39(1.34–1.44)	< 0.001	1.14(1.10–1.19)	< 0.001	1.23(1.17–1.28)	< 0.001
65-	1.92(1.81–2.04)	< 0.001	1.48(1.39–1.57)	< 0.001	1.63(1.53–1.75)	< 0.001
**Region**						
West	1(Ref)		1(Ref)		1(Ref)	
Middle	1.37(1.35–1.40)	< 0.001	1.28(1.26–1.30)	< 0.001	1.37(1.34–1.41)	< 0.001
East	0.63(0.63–0.64)	< 0.001	0.67(0.66–0.68)	< 0.001	0.80(0.77–0.83)	< 0.001
**Marital status**						
Never married/ Divorced/widowed	1(Ref)		1(Ref)		1(Ref)	
Married or living with partner	1.36(1.34–1.38)	< 0.001	1.36(1.34–1.39)	< 0.001	1.35(1.33–1.38)	< 0.001
**Educational attainment**						
<=Middle school	1(Ref)		1(Ref)		1(Ref)	
High school or equivalent	1.12(1.11–1.14)	< 0.001	1.13(1.11–1.15)	< 0.001	1.14(1.12–1.16)	< 0.001
College	1.21(1.19–1.23)	< 0.001	1.24(1.21–1.26)	< 0.001	1.26(1.23–1.28)	< 0.001
>College	1.11(1.02–1.21)	.020	1.25(1.14–1.37)	< 0.001	1.31(1.20–1.44)	< 0.001
**Household registration status**						
Rural	1(Ref)		1(Ref)		1(Ref)	
Urban	1.18(1.16–1.20)	< 0.001	1.09(1.07–1.11)	< 0.001	1.12(1.10–1.14)	< 0.001
**Length of migration**						
0–5	1(Ref)		1(Ref)		1(Ref)	
6–10	1.15(1.18–1.16)	< 0.001	1.14(1.12–1.16)	< 0.001	1.17(1.15–1.19)	< 0.001
> = 11	1.10(1.08–1.12)	< 0.001	1.12(1.10–1.15)	< 0.001	1.16(1.14–1.19)	< 0.001
**Reasons of migration**						
Studying/family issues	1(Ref)		1(Ref)		1(Ref)	
Working or business	0.74(0.73–0.75)	< 0.001	0.83(0.82–0.85)	< 0.001	0.87(0.85–0.90)	< 0.001
**Rage of migration**						
In province	1(Ref)		1(Ref)		1(Ref)	
Out of province	0.60(0.59–0.61)	< 0.001	0.72(0.71–0.73)	< 0.001	0.57(0.56–0.58)	< 0.001
Age×Sex					0.96(0.95–0.97)	< 0.001
Region×Reasons of migration					0.96(0.94–0.97)	< 0.001
Region×Rage of migration					1.29(1.27–1.31)	< 0.001
Length of migration×Household registration status					0.97(0.95–0.99)	.015
Length of migration×Educational attainment					0.98(0.96–0.99)	< 0.001

Compared with those who migrating for 0–5 years, migrants migrating for 6–10 years (aOR = 1.14; 95% CI,1.12–1.16), 11 and above years (aOR = 1.12; 95% CI,1.10–1.15) had a higher likelihood to establish health records. Compared with those who migrating for the reason of studying or family issues, migrants migrating for working or business (aOR = 0.83; 95% CI,.82–.85) were less likely to establish health records. Compared with those who migrating in province, migrants migrating out of province (aOR = 0.72; 95% CI,.71–.73) were less likely to establish health records.

In the results of model 1, the effects of age, region and length of migration on the establishment rate of health records are different from the assumptions. This shows that the impact of these three variables on the establishment rate of health records may be affected by other factors, so we need to add interaction terms to further test the impact of these three variables on the establishment rate of health records. Model 2 of multivariable analysis indicated that the interaction terms of age and gender, region and reasons of migration, region and rage of migration, length of migration and household registration status, together with length of migration and educational attachment were related to the increased establishment rate of health records, which means that age, region and length of migration not only exists main effects on the establishment rate of health records, but also conditional effects.

## Discussion

To our knowledge, this is the first study using national migrants-based data to examine the sociodemographic disparities in the establishment rate of health records among migrants in China. Our research reveals that the establishment rate of health records among the migrants differed in various sociodemographic characteristics, and heterogeneity existed in establish rate of a certain characteristic.

In terms of sociodemographic characteristics, we found that establishment rate of health records of female was higher than that of male’s. As far as the effect of age on the establishment rate of health records, it is generally believed that the older migrants tend to establish health records, which is not completely consistent with the results of model 1. This may be due to the influence of sex. Therefore, we add the interaction term of age and sex to test whether the impact of age on the establishment rate of health records is affected by sex, and sex truly had an impact on the effect of age on the establishment rate of health records. But from the overall trend, compared with young immigrants, older immigrants were more likely to establish health records, which was in line with the previous research. This may be the result of the fact that older people had a higher risk of having chronic diseases and worse health status, so they had a stronger motivation to seek health services. In addition, China’s BPHS regards the elderly over the age of 65 as a key service group, which may also be the reason for the higher establishment rate of health records among older people.

Compared with the west region, the migrants in the middle region were more likely to establish health records, and the immigrants in the east region were least likely to establish health records. In China, there are significant differences in the economic development of the east, middle and west region. It is generally believed that the economic development of the east region is better than that of the middle and the west region. In the views of the impact of economic development on the utilization of BPHS or health services in the previous studies [26, 27], the establishment rate of health records in China should be highest in the east region, followed by the middle and the west region. But the results of model 1 are inconsistent with this assumption. Due to the high economy development level in the east region, a large number of migrants cross provinces to work in the east region. Therefore, maybe they are less willing to establish health records due to lack of understanding of local health services.

Based on this, we added interaction terms between region and reasons of migration, as well as region and rage of migration to further explore the impact of economic development, which is represented by regions, on the establishment rate of health records. The results showed that there existed regional disparities of the establishment of health records of migrants in China, and the effect of region on the establishment of health records was influenced by migrating related factors, reasons of migration and rage of migration, which indicates a more complicated way to understand the disparities of the establishment rate of health records of migrants in China.

The reason might be that in China, the east part of the country had the most migrants, and the workforce of primary healthcare centers was limited. The professional staff of primary healthcare centers did not have enough time and energy to provide high-quality and enough health services. At the same time, during interviews with the staff of primary healthcare centers, we found that although the remuneration for providing services to each resident was increasing year by year, for some areas with inconvenient transportation, these remunerations may be less than the cost of providing services, which reduced their enthusiasm of providing services [13].

Compared with the migrants with rural household registration, the possibility of establishing health records was higher for migrants with urban household registration, which is in line with the previous researches [13, 20, 24]. Although in recent years, China is reforming its household registration system and gradually eliminating the differences in public services between urban and rural areas, China’s dual household registration system still affects residents’ utilization of public health services. Moreover, the household registration system not only has main effect, but also conditional effects on the establishment of health records, as well as the utilization of other public services [20]. The higher the degree of education was, the higher the possibility of establishing health records was. The effect of educational attainment on the establishment of health records was also influenced by length of migration, and the reason was probably that education and health literacy assisted migrants in gaining a better understanding of the advantages of establishing health records [28–30].

As far as migrating characteristics, some studies show that the longer migrants stay at the destination, the higher the degree of social integration and the establishment rate of health records [7, 18], which is different from the results in model 1. We introduce the interaction terms between length of migration and household registration status, together with length of migration and educational attachment to further explore whether the impact of length of migration on the establishment rate of health records is influenced by the household registration status and educational attachment. It turns out that the effect of length of migration on the establishment rate of health records was influenced by the household registration status educational attainment. This maybe because that the longer migrants stayed in the place of residence, the more familiar they were with the local health system, and higher the residents’ awareness of seeking health services. Compared with migrants who were migrating for the reason of studying or family issues, people who intended to work or do business were less likely to establish health records. Compared with those who migrated within the province, those who migrated outside the province were less likely to establish health records.

In terms of improving the access of health services, the provision of BPHS is an important measure of the Chinese government. All costs of providing related services are borne by the government, and residents do not have to pay for themselves. In recent years, the Chinese government has also carried out other reforms, such as “family physicians services”, which aims to reduce the waiting time for residents to obtain health services and the economic burden of residents improve the efficiency of health services, increase the satisfaction of residents, and improve their health through signing contracts with family physician of primary healthcare centers. Whether it is BPHS or “family physicians service”, migrants can get the same services as local residents. However, our research shows that the establishment rate of health records among the migrants remain at a relatively low level, which may hinder the improvement of health equity.

The reasons for the low establishment rate of health records can be analyzed from two aspects. From the supply side, China’s health resources are distributed in the shape of “inverted pyramid”, which means that health resources are concentrated in secondary and tertiary hospitals. Community health centers have relatively limited health resources. The lack of human resources, coupled with heavy workload of providing BPHS, leading to burnout and high work stress of professional staff, directly affect the provision of BPHS [5]. From the demand side, although BPHS have been implemented for many years, due to the lack of project publicity and health education, migrants’ awareness of obtaining BPHS is still relatively low, thus they rarely come to community health centers for BPHS [31].

The health of the migrants is an important social issue and public policy issue. The establishment of health records strengthens the health management of the migrants, which is an effective way to improve the health of the migrants. Therefore, relevant departments should take actions to promote community health centers to provide more effective and high-quality services, and enhance residents’ awareness of health management. Measures should be taken to strengthen health education and health literacy of migrants. At the same time, relative departments should continue to invest in primary healthcare centers to improve their ability to provide better services to meet the needs of residents and migrants.

Our research has several limitations. First, There may be recall bias when participants recalled whether he/she had established a health record, which leads to the underestimation of establishment rate of health records; Second, other factors that may affect the establishment rate of health records (such as family economic status) have not been investigated in the survey, so these variables cannot be included in this study; The effect of family economic status, for example, household income, has different effects on the establishment rate of health records [13, 24]. Since the economic development status of the east,the middle, the west regions are different, we believe that the region variable can partly refer to the level of economic development. But the effect of household income need further exploring. Third, because this survey is not a continuous survey, this study cannot analyze the long-term trend of establishment rate of health records. However, through 3 years of national survey data, this study provides important basic information and scientific evidence for improving the accessibility of basic public health services and health equity.

## Conclusion

Sociodemographic disparities existed in the establishment rate of health records among China’s migrants. The associated migrating characteristics, including length of migration, reasons of migration and rage of migration, also had impact on the establishment rate of health records. The study revealed heterogeneity in the establishment rate of health records in the subgroups, which policies should take into account. In addition to the reforms of the supply side, the reforms should also focus on the demand side, intending to improve the migrants’ awareness of health management.

## Supplementary Information

**Additional file 1: Supplemental Table 1**. Items of Basic Public Health Services and prices from 2009 to 2020.

## Data Availability

The dataset supporting the conclusions of this article was acquired at https://www.ncmi.cn/phda/dataDetails.do?id=CSTR:A0006.11.A000T.201906.000225.

## Abbreviations

BPHS: Basic Public Health Services

## References

[1] Chen T, Wang Y, Luo X, Rao Y, Hua L (2018). Correction to: Inter-provincial inequality of public health services in China: the perspective of local officials' behavior. Int J Equity Health.

[2] Wang Z, Ao Q, Luo Y, Wang Q, Lu Z, Liu J (2019). Estimating the costs of the national basic public health services in Zhuhai, China, through activity-based costing: a cross-sectional study. BMJ Open.

[3] Meng X (2019). Does a Different Household Registration Affect Migrants' Access to Basic Public Health Services in China?. Int J Environ Res Public Health.

[4] Guo L, Bao Y, Li S, Ma J, Sun W (2018). Quality analysis and policy recommendations on the utilization of community basic public health services in urban and suburban Shanghai from 2009 to 2014. Environ Sci Pollut Res Int.

[5] Zhang J, Lin S, Liang D, Qian Y, Zhang D, Hou Z (2017). Public Health Services Utilization and Its Determinants among Internal Migrants in China: Evidence from a Nationally Representative Survey. Int J Environ Res Public Health.

[6] Zhu Z, Guo M, Petrovsky DV, Dong T, Hu Y, Wu B (2019). Age and regional disparity in HIV education among migrants in China: migrants population dynamic monitoring survey, 2014-2015. Int J Equity Health.

[7] Liang J, Shi Y, Osman M, Shrestha B, Wang P (2020). The association between social integration and utilization of essential public health services among internal migrants in China: a multilevel logistic analysis. Int J Environ Res Public Health.

[8] Zheng L, Hu R, Dong Z, Hao Y (2018). Comparing the needs and utilization of health services between urban residents and rural-to-urban migrants in China from 2012 to 2016 11 medical and health sciences 1117 public health and health services. BMC Health Serv Res.

[9] Zhang X, Yu B, He T, Wang P (2018). Status and determinants of health services utilization among elderly migrants in China. Glob Heal Res Policy.

[10] Griffiths SM, Tang JL (2011). Healthcare reform in China and the challenges for public health education. Public Health.

[11] Hou J, Wang Z, Liu X (2018). Public health education at China’s higher education institutions: a time-series analysis from 1998 to 2012. BMC Public Health.

[12] Jin Y, Wang H, Wang D, Yuan B (2019). Job satisfaction of the primary healthcare providers with expanded roles in the context of health service integration in rural China: a cross-sectional mixed methods study. Hum Resour Health.

[13] Liu J, Mao Y (2019). Rural resident experience on national basic public health services: A cross-sectional survey in 10 western provinces of china. Healthcare (Basel).

[14] Yang L, Sun L, Wen L (2016). Financing strategies to improve essential public health equalization and its effects in China. Int J Equity Health.

[15] Chen T, Wang Y, Luo X, Rao Y, Hua L (2018). Inter-provincial inequality of public health services in China: the perspective of local officials’ behavior. Int J Equity Health.

[16] Shao S, Wang M, Jin G, Zhao Y, Lu X, Du J (2018). Analysis of health service utilization of migrants in Beijing using Anderson health service utilization model. BMC Health Serv Res.

[17] Yin D, Wong ST, Chen W (2015). A model to estimate the cost of the National Essential Public Health Services Package in Beijing, China. BMC Health Serv Res.

[18] Hou Z, Lin S, Zhang D (2017). Social capital, neighbourhood characteristics and utilisation of local public health services among domestic migrants in China: a cross-sectional study. BMJ Open.

[19] Jing Z, Wang Y, Ding L, Tang X, Feng Y, Zhou C (2019). Effect of social integration on the establishment of health records among elderly migrants in China: a nationwide cross-sectional study. BMJ Open.

[20] Wang Z, Wu Q, Ming J (2020). The relationship between homeownership and the utilization of local public health services among rural migrants in China: a Nationwide cross-sectional study. Front Public Heal.

[21] Song X, Zou G, Chen W, Han S, Zou X, Ling L (2017). Health service utilisation of rural-to-urban migrants in Guangzhou, China: does employment status matter?. Trop Med Int Heal.

[22] Guo M, Zhu Z, Dong T, Mi H, Wu B (2019). Provincial and age disparity on chronic disease education among migrants in China: the migrants population dynamic monitoring survey. Inquiry..

[23] Tang D, Wang J (2021). Basic Public Health Service Utilization by Internal Older Adult Migrants in China. Int J Environ Res Public Health.

[24] Qian Y, Ge D, Zhang L, Sun L, Li J, Zhou C (2018). Does Hukou origin affect establishment of health records in migrant inflow communities? A nation-wide empirical study in China. BMC Health Serv Res.

[25] Long C, Tang S, Wang R (2020). The migrating mediators and the interaction associated with the use of essential public health services: a cross-sectional study in Chinese older migrants. BMC Geriatr.

[26] Lam KKF, Johnston JM (2012). Health insurance and healthcare utilisation for Shenzhen residents: a tale of registrants and migrants?. BMC Public Health.

[27] Flegar V, Dalli M, Toebes B (2016). Access to preventive health Care for Undocumented Migrants: a comparative study of Germany, the Netherlands and Spain from a human rights perspective. Laws..

[28] Cai X, Yang F, Bian Y (2019). Gap analysis on hospitalized health service utilization in floating population covered by different medical insurances - - - case study from Jiangsu Province, China. Int J Equity Health.

[29] Curtis P, Thompson J, Fairbrother H (2018). Migrant children within Europe: a systematic review of children’s perspectives on their health experiences. Public Health.

[30] Schilte AF, Portegijs PJM, Blankenstein AH (2001). Randomised controlled trial of disclosure of emotionally important events in somatisation in primary care. Br Med J.

[31] Hou Z, Lin S, Zhang D (2017). Social capital, neighbourhood characteristics and utilisation of local public health services among domestic migrants in China: a cross-sectional study. BMJ Open.

