# Supplementary material for: Sociodemographic disparities in the establishment of health records among 0.5 million migrants from 2014 to 2017 in China: a nationwide cross-sectional study
Source: Int J Equity Health. 2021 Dec 2;20:250. doi: 10.1186/s12939-021-01584-2 (PMC8638552; doi:10.1186/s12939-021-01584-2)
Supplement: Supplementary file 1 — Additional file 1: Supplemental Table 1. Items of Basic Public Health Services and prices from 2009 to 2020. [file 12939_2021_1584_MOESM1_ESM.docx]

**Supplemental Table 1** Items of Basic Public Health Services and prices from 2009-2020

| **Year** | **Items of BPHS** | **Service price per person(RMB)** |
| --- | --- | --- |
| 2009 | Health record management, health education, vaccination, children health management, maternal health management, elderly health management, chronic disease management (health management of hypertensive patients, health management of type 2 diabetes patients), management of patients with severe mental illness, reporting of infectious diseases and public health emergencies | 15 |
| 2011 | Add "Health supervision Assistance" | 25 |
| 2013 | Same as 2011 | 30 |
| 2014 | Add "Chinese Medicine Related services" | 35 |
| 2015 | Add "Health Management for Tuberculosis Patients" | 40 |
| 2016 | Same as 2016 | 45 |
| 2017 | Same as above | 50 |
| 2018 | Same as above | 55 |
| 2019 | Same as above | 69 |
| 2020 | Same as above | 74 |
